# Incidence, consequences, and predictors of serious chemotherapy‐induced thrombocytopenia in nasopharyngeal carcinoma

**DOI:** 10.1002/cam4.6062

**Published:** 2023-05-22

**Authors:** Lu‐Lu Zhang, Xi Chen, Ying‐Ying Huang, Chi‐Xiong Liang, Meng‐Yun Qiang, Zhuo‐Chen Cai, Ze‐Jiang Zhan, Ying Deng, Jia‐Yu Zhou, Hao‐Yang Huang, Xiang Guo, Xing Lv

**Affiliations:** ^1^ Department of Nasopharyngeal Carcinoma, Sun Yat‐sen University Cancer Center, State Key Laboratory of Oncology in South China, Guangdong Key Laboratory of Nasopharyngeal Carcinoma Diagnosis and Therapy Collaborative Innovation Center for Cancer Medicine Guangzhou People's Republic of China; ^2^ Department of Head and Neck Radiotherapy, The Cancer Hospital of the University of Chinese Academy of Sciences (Zhejiang Cancer Hospital), Institute of Basic Medicine and Cancer (IBMC) Chinese Academy of Sciences Hangzhou People's Republic of China

**Keywords:** chemotherapy, nasopharyngeal carcinoma, predictor, thrombocytopenia

## Abstract

**Objectives:**

This study aimed to investigate the incidence, consequences, and predictors of serious chemotherapy‐induced thrombocytopenia (CIT) in nasopharyngeal carcinoma (NPC).

**Materials and Methods:**

We retrospectively reviewed the clinical records of patients with NPC between 2013 and 2015. Multivariate Cox proportional hazards regression model and propensity score matching were used to estimate the effect of serious CIT on overall survival. Univariate and multivariate logistic regression analyses were applied to identify the predictors of serious CIT.

**Results and Conclusion:**

The incidence of serious CIT was 5.21% in patients with NPC. Patients who experienced serious thrombocytopenia had a worse long‐term prognosis, while the difference in short‐term survival rate was slight. Chemotherapy regimens of gemcitabine and platinum, 5‐fluorouracil and platinum, taxane and platinum, serum potassium ion concentration, serum lactate dehydrogenase levels, platelet count, red blood cell count, and estimated glomerular filtration rate were predictors of serious CIT.

## 1 INTRODUCTION

In 2020, there were 133,354 new cases of nasopharyngeal carcinoma (NPC), accounting for 0.7% of the total cases of 36 cancers.^1^ Radiotherapy and chemotherapy are the main treatment modalities for patients with NPC. A number of clinical trials have indicated that concurrent chemotherapy and induction chemotherapy benefit locally advanced NPC compared to radiotherapy alone.^2^ Induction chemotherapy containing platinum, for two to four times, for instance, gemcitabine and cisplatin, docetaxel and cisplatin, cisplatin and 5‐fluorouracil, docetaxel plus cisplatin and 5‐fluorouracil were recommended.^3^ The majority of patients with NPC require chemotherapy because they are likely to be locoregionally advanced when they seek medical care. Several studies have shown that radiotherapy for neck and head cancers does not result in serious thrombocytopenia.^4^, ^5^, ^6^ Therefore, it can be deduced that chemotherapy induced serious thrombocytopenia in the treatment of NPC. One study reported that chemotherapy is a risk factor for thrombocytopenia during radiotherapy.^7^ Chemotherapy‐induced thrombocytopenia (CIT), which has a high cost, leads to reduced, delayed, changed, or discontinued chemotherapy dose.^8^, ^9^, ^10^ A platelet count ≤10 × 10^9^/L increases the likelihood of major bleeding.^11^ CIT affects not only chemotherapy but also radiotherapy. Therefore, it is necessary to investigate the incidence, consequences, and predictors of serious CIT.

## 2 PATIENTS AND METHODS

### 2.1 Patients

We retrospectively reviewed the records of newly diagnosed patients with non‐metastatic NPC from the Sun Yat‐Sen University Cancer Center (SYSUCC) between 2013 and 2015. The inclusion criteria were as follows: (1) aged from 18 to 75 years when diagnosed; (2) pretreatment white blood cell (WBC) count ≥4 × 10^9^/L and platelet count ≥100 × 10^9^/L; (3) received radiotherapy and platinum‐containing chemotherapy; (4) without other malignant diseases. The exclusion criterion was a change in the chemotherapy regimen for reasons other than thrombocytopenia. According to the Common Terminology Criteria for Adverse Events (CTCAE version 5.0), thrombocytopenia grade 3 is defined as platelet count between 25 × 10^9^/L and 50 × 10^9^/L, and grade 4, platelet count <25 × 10^9^/L.^12^ We defined serious CIT as grade 3–4 thrombocytopenia occurring within 120 days from the start of treatment. This retrospective study was approved by the institutional ethics review board of the SYSUCC (approval number B2022‐684‐01).

### 2.2 Statistical analyses

To analyze the baseline characteristics of patients, the Mann–Whitney–Wilcoxon test was used for count data and the chi‐squared test was used for categorical data. Univariate and multivariate Cox proportional hazards regression models were used to estimate the effect on overall survival (OS), and Schoenfeld residuals were calculated to test the proportional hazards assumption. To further demonstrate the above result, survival analysis was evaluated using the Kaplan–Meier method and examined with the log‐rank test after adjusting for confounding factors using propensity score matching (PSM). Univariate and multivariate logistic regression analyses were applied to identify the predictors of serious CIT. To find the threshold of predictors, receiver operating characteristic (ROC) curve analysis was performed, and the area under the curve (AUC) was calculated. OS was defined as the time from diagnosis to death from any cause or the date of last follow‐up for patients alive or lost to follow‐up. All statistical analyses were conducted using R (version 4.1.3) with tableone, MatchIt, survminer, survival, rms, and pROC packages.^13^, ^14^, ^15^, ^16^, ^17^, ^18^

## 3 RESULTS

### 3.1 Baseline characteristics

A total of 27 patients were excluded from analyses due to a change in chemotherapy regimen for reasons other than thrombocytopenia. The reasons for a change of chemotherapy were as follows: poor response to the first‐line chemotherapy (*n* = 12), serious neutropenia (*n* = 4), request for a shorter hospital stay (*n* = 3), and unknown reasons (*n* = 8). Finally, a total of 3933 patients were included in this study. Table 1 presents the baseline characteristics of patients. The incidence of serious CIT was 5.21% (*n* = 205) within 120 days from the start of treatment. The incidences of serious CIT among patients receiving platinum alone, gemcitabine and platinum (GP), platinum and 5‐fluorouracil (PF), taxane and platinum (TP), taxane plus platinum and 5‐fluorouracil (TPF) were 2.66%, 13.97%, 10.17%, 5.02%, and 3.72%.

**TABLE 1:** Baseline characteristics before and after PSM.

	Before PSM			After PSM		
	Without serious CIT (*n* = 3728)	With serious CIT (*n* = 205)	*p*	Without serious CIT (*n* = 587)	With serious CIT (*n* = 201)	*p*
Age (mean [SD] years)	44.87 (10.45)	48.47 (10.94)	<0.001	47.63 (10.49)	48.16 (10.82)	0.565
LDH (%)						
LDH < 245 U/L	3428 (92.0)	176 (85.9)	0.003	516 (87.9)	175 (87.1)	0.851
LDH ≥ 245 U/L	300 (8.0)	29 (14.1)		71 (12.1)	26 (12.9)	
Sex (%)			1.000			0.441
Male	2757 (74.0)	152 (74.1)		450 (76.7)	148 (73.6)	
Female	971 (26.0)	53 (25.9)		137 (23.3)	53 (26.4)	
T (%)			0.071			0.623
1	409 (11.0)	16 (7.8)		51 (8.7)	16 (8.0)	
2	512 (13.7)	18 (8.8)		44 (7.5)	168 (9.0)	
3	2111 (56.6)	128 (62.4)		349 (59.5)	126 (62.7)	
4	696 (18.7)	43 (21.0)		143 (24.4)	41 (20.4)	
*N* (%)			0.057			0.521
0	371 (10.0)	14 (6.8)		54 (9.2)	14 (7.0)	
1	1602 (43.0)	75 (36.6)		224 (38.2)	72 (35.8)	
2	1152 (30.9)	77 (37.6)		216 (36.8)	76 (37.8)	
3	603 (16.2)	39 (19.0)		93 (15.8)	39 (19.4)	
Stage (%)			0.001			0.955
I	56 (1.6)	3 (1.5)		6 (1.0)	3 (1.5)	
II	442 (11.9)	6 (2.9)		18 (3.1)	6 (3.0)	
III	2008 (53.9)	120 (58.5)		350 (59.6)	118 (58.7)	
IVa	1219 (32.7)	76 (37.1)		213 (36.3)	74 (36.8)	
Chemotherapy regimen (%)			<0.001			0.908
Platinum alone	1462 (39.2)	40 (19.5)		117 (19.9)	40 (19.9)	
GP	117 (3.1)	19 (9.3)		39 (6.6)	17 (8.5)	
PF	733 (19.7)	83 (40.5)		242 (41.2)	81 (40.3)	
TP	587 (15.7)	31 (15.1)		100 (17.0)	40 (15.4)	
TPF	829 (22.2)	32 (15.6)		89(15.2)	32 (15.9)	

*Note*: Patients with serious CIT are patients who experienced serious thrombocytopenia within 120 days from the day of diagnosis.

Abbreviations: CIT, chemotherapy‐induced thrombocytopenia; GP, gemcitabine and platinum; LDH, lactate dehydrogenase; Platinum alone means that the chemotherapy regimen only includes platinum; PF, 5‐fluorouracil and platinum; PSM, propensity score matching; T, tumor; N, node; and stage were regraded based on the eighth edition of AJCC staging system for nasopharyngeal carcinoma; TP, taxane and platinum; TPF, taxane plus platinum and 5‐fluorouracil.

### 3.2 Cox regression survival analyses

**FIGURE 1:**
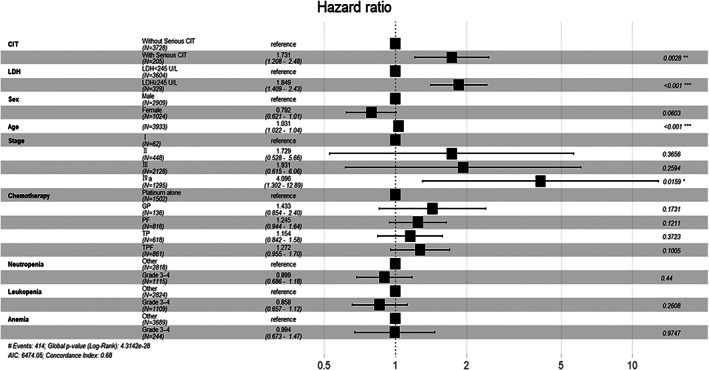
Forest plot of multivariate Cox proportional hazards regression model. CIT, chemotherapy‐induced thrombocytopenia; GP, gemcitabine and platinum; LDH, lactate dehydrogenase; TP, taxane and platinum; TPF, taxane plus platinum and 5‐fluorouracil. *p < 0.05, **p < 0.01,***p < 0.001.

OS was analyzed using univariate and multivariate Cox proportional hazards regression models. CIT was a risk factor in the univariate Cox regression analysis (hazard ratio [HR], 2.07; 95% confidence interval [CI], 1.49–2.87; *p* < 0.001). The multivariate Cox model was adjusted for factors including age, stage, chemotherapy regimen, sex, pretreatment serum lactate dehydrogenase (LDH), and whether patients experienced grade 3–4 CIT, neutropenia, leukopenia, or anemia within 120 days from the start of treatment. A higher LDH level was LDH ≥245 U/L.^19^ Grade 3–4 neutropenia was defined as neutrophil count <1.0 × 10^9^/L and grade 3–4 leukopenia was defined as WBC count <2.0 × 10^9^/L. Grade 3–4 anemia was defined as hemoglobin level <80 g/L.^12^ All the variables conformed to proportional hazards, which were tested using Schoenfeld residuals. The results of the multivariate Cox regression model are shown in Figure 1. According to the outcome, patients who experienced serious CIT (HR, 1.731; 95% CI, 1.208–2.48; *p* = 0.0028), a higher LDH (LDH ≥ 245 U/L) level (HR, 1.849; 95% CI, 1.409–2.43; *p* < 0.001), age (HR, 1.031; 95% CI, 1.022–1.04; *p* < 0.001), and stage IVa (HR, 4.096; 95% CI, 1.302–12.89; *p* = 0.0159) were statically significant risk factors for OS. Sex, chemotherapy regimen, and Grade 3–4 neutropenia, leukopenia, and anemia were non‐significantly associated with OS (*p* > 0.05).

### 3.3 Survival analyses after PSM

To further prove the reliability of the conclusion that serious CIT was a risk factor for OS, PSM was applied to balance the other variables, after which the Kaplan–Meier method and log‐rank test were used. Variables, such as age, stage, chemotherapy regimen, and LDH level were included in PSM. Patients with serious CIT were matched to patients without serious CIT for a 3:1 nearest‐neighbor PSM with a caliper level of 0.05.

A total of 587:201 patients (2.92:1) were included after PSM. The patient characteristics were balanced. Table 1 presents the results. The differences between the variables in the two groups were not significant (*p* > 0.05).

**FIGURE 2:**
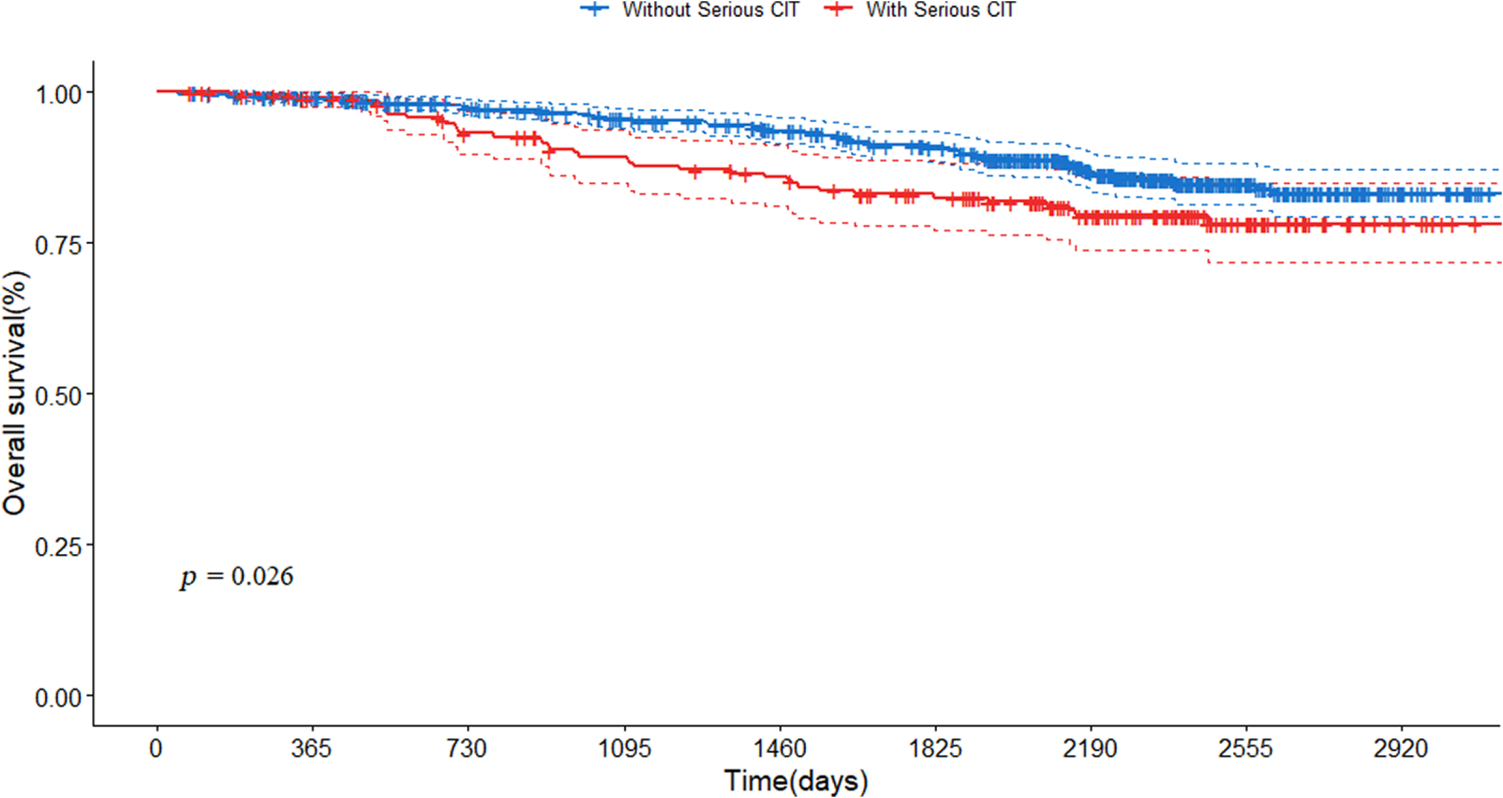
Kaplan–Meier curves after PSM. CIT, chemotherapy‐induced thrombocytopenia; PSM, propensity score matching.

The median follow‐up time, calculated using the reverse Kaplan–Meier method, was 2298 days (95% CI, 2259–2348). The 5‐year OS of patients with serious CIT was 82.5% (95% CI, 77.1%–88.2%), which was significantly lower than that of patients without serious CIT, with a 5‐year OS of 90.9% (95% CI, 88.4%–93.4%). The 1‐year OS of patients with serious CIT was 98.98% (95% CI, 97.58%–100.0%), and patients without serious CIT was 99.1% (95% CI, 98.4%–99.9%). Survival curves were described by Kaplan–Meier curves and compared using the log‐rank test, as shown in Figure 2. The differences in the survival curves of patients with serious CIT and patients who did not were statistically different (*p* < 0.05).

### 3.4 Effects on treatment

To find the underlying cause of decreased OS in patients with CIT, we measured the effects of CIT on chemotherapy and radiotherapy.

Six patients changed the chemotherapy regimen because of CIT and three patients died. 148 patients finished the planned chemotherapy treatment. Fifty‐four patients had chemotherapy dose reduction or complete chemotherapy stop. Patients who had chemotherapy reduction or stop had poorer OS (75.9% vs. 83.1%). However, the decrease in OS was statistically insignificant before and after adjusting for important factors (*p* > 0.05). Sixty‐four patients received chemotherapy after CIT and 50% (*n* = 32) patients had delays in chemotherapy (interval time ≥ 28) due to CIT. The difference in OS was insignificant between patients with and without delays in chemotherapy before and after adjusting for important factors (81.2% vs. 65.6%; *p* > 0.05). Three patients had unplanned radiotherapy interruption due to side effects other than CIT. Tree patients had incomplete records of radiotherapy duration. One patient who stopped radiotherapy due to CIT received a total dose of 28 Gy for the gross tumor volume and died within 4 years. 172 patients continued radiotherapy despite experiencing CIT. Twenty‐nine patients had an interruption of radiotherapy due to CIT. A significantly longer radiotherapy treatment time (RTT) was observed in patients with radiotherapy interruption (44.74 vs. 52.48 days; *p* < 0.001). However, the effect of radiotherapy interruption on OS was insignificant before and after adjusting for important factors (82.8% vs. 81.4%; *p* > 0.05). Table 2 presents the results.

**TABLE 2:** Effects on treatment.

	Patients with serious CIT	Duration of radiotherapy		Overall survival	
		Day (mean [SD] days)	*p*	HR (95% CI)^a^	*p*
Chemotherapy dose^b^					
Planned treatment	148	45.84 (5.17)	0.429	Reference	0.170
Chemotherapy reduction or stop	54	45.88 (5.55)		1.60 (0.818–3.13)	
Chemotherapy delay^c^					
Interval time ≥28 days	32	45.41 (4.23)	0.630	Reference	0.171
Interval time <28 days	32	44.87 (4.55)		0.484(0.171–1.37)	
Radiotherapy^d^					
Without interruption	172	44.74 (3.75)	**<0.001**	Reference	0.626
Interruption of radiotherapy	29	52.48 (6.04)		0.79 (0.299–2.07)	

Abbreviation: CIT, chemotherapy‐induced thrombocytopenia.

Bold values indicate statistically significant (*p* < 0.05).

^a^ HR was calculated using a multivariate Cox proportional hazards regression model which was adjusted for important factors, including sex, age, stage, and lactate dehydrogenase level.

^b^ Patients with a change of chemotherapy were excluded (*n* = 6).

^c^ Only patients who had chemotherapy after CIT were included.

^d^ Patients with incomplete radiotherapy records, radiotherapy stop, and unplanned interruption due to side effects other than CIT were excluded (*n* = 7).

### 3.5 Predictors of CIT

To investigate the predictors of serious CIT, we performed univariate and multivariate logistic regression analyses with 15 variables, including age, sex, stage, chemotherapy regimen, body mass index (BMI), and pretreatment data, including LDH, white blood cell (WBC) count, red blood cell (RBC) count, lymphocyte (LYM) count, platelet (PLT) count, absolute neutrophil count (ANC), serum indirect bilirubin (IBIL), serum potassium ion concentration (K^+^), serum sodium ion concentration (Na^+^), and estimated glomerular filtration rate (eGFR). The Chronic Kidney Disease Epidemiology Collaboration creatinine–cystatin C equation was applied to assess eGFR.^20^ Table 3 presents the results.

**TABLE 3:** Odds ratios (ORs) and *p*‐values of univariate and multivariate logistic regression analyses.

	Univariate		Multivariate		Final multivariate logistic regression	
	OR (95% CI)	*p*	OR (95% CI)	*p*	OR (95% CI)	*p*
Age (years)	1.03 (1.02–1.05)	**<0.0001**	1.01 (1.00–1.03)	0.1659		
BMI (kg/m^2^)	0.96 (0.91–1.00)	**0.0470**	0.96 (0.92–1.01)	0.0999		
WBC (10^9^/L)	0.93 (0.86–1.01)	0.0814	0.81 (0.43–1.45)	0.4864		
RBC (10^12^/L)	0.66 (0.50–0.87)	**0.0027**	0.70 (0.51–0.96)	**0.0320**	0.62 (0.46–0.82)	**0.0012**
LYM (10^9^/L)	0.84 (0.67–1.07)	0.1553	1.33 (0.65–2.79)	0.4421		
ANC (10^9^/L)	0.94 (0.86–1.03)	0.1827	1.31 (0.71–2.51)	0.4030		
PLT (10^9^/L)	0.99 (0.99–0.99)	**<0.0001**	0.99 (0.99–0.99)	**<0.0001**	0.99 (0.99–0.99)	**<0.0001**
IBIL (μmol/L)	1.01 (0.97–1.06)	0.5069	1.01 (0.96–1.05)	0.7846		
K^+^ (mmol/L)	1.51 (1.01–2.27)	**0.0442**	1.60 (1.04–2.46)	**0.0322**	1.57 (1.03–2.39)	**0.0368**
Na^+^ (mmol/L)	1.04 (0.97–1.10)	0.2886	1.01 (0.95–1.08)	0.7192		
eGFR (mL/min/1.73 m^2^)	0.97 (0.96–0.98)	**<0.0001**	0.98 (0.97–0.99)	**0.0010**	0.98 (0.97–0.99)	**<0.0001**
LDH						
LDH < 245 U/L	Reference		Reference		Reference	
LDH ≥ 245 U/L	1.88 (1.25–2.84)	**0.0025**	1.76 (1.11–2.71)	**0.0123**	1.83 (1.17–2.77)	**0.0063**
Sex						
Male	Reference		Reference			
Female	0.99 (0.72–1.36)	0.9512	1.23 (0.83–1.81)	0.2949		
Stage						
I	Reference		Reference			
II	0.27 (0.07–1.10)	0.0668	0.25 (0.06–1.25)	0.0614		
III	1.18 (0.36–3.80)	0.7875	0.84 (0.29–3.55)	0.7757		
IVa	1.23 (0.38–4.00)	0.7355	0.81 (0.28–3.48)	0.7403		
Chemotherapy regimen						
Platinum alone^a^	Reference		Reference		Reference	
GP	5.94 (3.33–10.58)	**<0.0001**	5.79 (3.11–10.48)	**<0.0001**	6.30 (3.41–11.31)	**<0.0001**
PF	4.14 (2.81–6.10)	**<0.0001**	4.11 (2.76–6.22)	**<0.0001**	4.51 (3.05–6.76)	**<0.0001**
TP	1.93 (1.20–3.12)	**0.0071**	1.88 (1.14–3.08)	**0.0127**	2.05 (1.25–3.33)	**0.0041**
TPF	1.41 (0.88–2.26)	0.1534	1.42 (0.86–2.32)	0.1660	1.52 (0.94–2.46)	0.0859

Abbreviation: ANC, absolute neutrophil count; BMI, body mass index; eGFR, estimated glomerular filtration rate; GP, gemcitabine and platinum; IBIL, serum indirect bilirubin; K^+^, serum potassium ion concentration; LDH, lactate dehydrogenase; LYM, lymphocyte count; Na^+^, serum sodium ion concentration; PF, 5‐fluorouracil and platinum; PLT, platelet count; RBC, red blood cell count; TP, taxane and platinum; TPF, taxane plus platinum and 5‐fluorouracil; WBC, white blood cell count.

Bold values indicate statistically significant (*p* < 0.05).

^a^ Platinum alone means that the chemotherapy regimen only includes platinum.

Variables with *p*‐value <0.05 in the previous multivariate logistic regression were included in the final multivariate logistic regression. The results of the final multivariate logistic regression, including RBC, PLT, K^+^, LDH, eGFR, and chemotherapy regimen are shown in Table 3. The variables were lowly collinear, as tested by the variance inflation factor (VIF < 1.8). It could conclude that GP (OR, 6.30; 95% CI, 3.41–11.31; *p* < 0.0001), PF (OR, 4.51; 95% CI, 3.05–6.76; *p* < 0.0001), and TP (OR, 2.05; 95% CI, 1.25–3.33; *p* = 0.0041) chemotherapy, K^+^ (OR, 1.57; 95% CI, 1.03–2.39; *p* = 0.0368), LDH (≥245 U/L) (OR, 1.83; 95% CI, 1.17–2.77; *p* = 0.0063), RBC count (OR, 0.62; 95% CI, 0.46–0.82; *p* = 0.0012), eGFR (OR, 0.98; 95% CI, 0.97–0.99; *p* < 0.0001), and PLT count (OR, 0.99; 95% CI, 0.99–0.99; *p* < 0.0001) were predictive factors of serious CIT. For clinical convenience, we determined the threshold of PLT count and eGFR using ROC curve analysis. The cut‐off value of PLT count was 228.75 × 10^9^/L (AUC, 0.662) and eGFR was 94.60 mL/min/1.73 m^2^ (AUC, 0.610).

## 4 DISCUSSION

Serious CIT has long been a hot topic. However, to our knowledge, our study is the first to report the effects of CIT on long‐term survival and the first to report that higher serum K^+^ was a risk factor for CIT.

Whether patients who experienced neutropenia or leukopenia had worse prognoses was controversial.^21^, ^22^, ^23^, ^24^, ^25^, ^26^, ^27^ Prior studies reported a worse prognosis in patients with anemia.^28^, ^29^ In our study, grade 3–4 neutropenia, leukopenia, and anemia were not prognostic factors. CIT was a risk factor for OS before and after adjusting the factors of grade 3–4 neutropenia, leukopenia, or anemia which might occur at the same time as CIT. According to Kaplan–Meier curves, the lines were close in the first year and the 1‐year OS of patients with serious CIT was 98.98%, and patients without serious CIT was 99.1%. We can infer that CIT will not significantly affect the short‐term survival rate. This might be due to the low probability of fatal bleeding because the methods used to treat CIT increase gradually. The 5‐year survival rates of the two groups were significantly different.

In our study, the RTT was prolonged in 29 patients with CIT. Previous studies reported a worse OS in patients with prolonged RTT whose threshold was 49–56 days.^30^, ^31^, ^32^ The average time of prolonged RTT was 52.48 days in our study. No significant difference in OS was observed in patients with prolonged RTT who had CIT in our study. The negative result was likely due to a brief radiotherapy stop which was shorter than 56 days. According to previous studies, cumulative cisplatin dose >200 mg/m^2^ improved survival.^33^, ^34^ In our study, 54 patients had chemotherapy dose reduction or complete chemotherapy stop due to CIT. The decrease in OS was insignificant compared with patients who experienced CIT and finished planned chemotherapy. Based on the results, we could conclude that neither the prolonged RTT nor the chemotherapy dose reduction and delays contribute to the decrease in OS. However, it was worth noting that the sample size was small in subgroup analyses. Further studies are needed to investigate the inner relationship between CIT and decreased OS, the effect of thrombopoietic agents on long‐term survival, and the microenvironment change when CIT occurs.

In a previous study, lower eGFR (<60 mL/min/1.73 m^2^) was a risk factor for CIT.^35^ In our study, higher eGFR was a protective factor in the univariate and multivariate regression models. The threshold of eGFR was 94.60 mL/min/1.73 m^2^ (AUC, 0.610), which meant the effect of eGFR on CIT also exists in patients with normal renal function. The mechanism might be that better renal function results in more chemotherapy drug excretion, and fewer side effects on megakaryocytes. We also found that eGFR was not a predictive factor of OS through multivariate Cox proportional hazards regression analysis. More chemotherapy drug excretion may not cause a worse prognosis. Further research on the relationship between eGFR and drug concentration in serum is needed to prove this hypothesis.

Our data demonstrated that higher serum K^+^ levels are associated with a higher risk of serious CIT. As already reported, the elevation of serum K^+^ is related to cell and platelet lysis.^36^ Previous studies have suggested that potassium supplementation can decrease platelet reactivity.^37^ Whether these theories can be applied to serious CIT requires further investigation. A higher level of LDH was a risk factor for CIT in diffuse large B‐cell lymphoma.^38^ Our study was the first to report that LDH was a risk factor for CIT in solid tumors. The molecular mechanisms for it warrant further research.

Previous studies found that lower RBC count and PLT count were associated with an increased risk of CIT.^39^, ^40^ We also proved that higher RBC and PLT were protective factors of CIT. Long‐term survival is affected by a serious CIT. This might be an explanation for the conclusion of previous studies that PLT <150 × 10^9^/L was associated with poor survival.^41^, ^42^ One study reported some genes associated with the proliferation of megakaryocytes related to CIT in patients with non‐small cell lung cancer who have received carboplatin and gemcitabine.^43^ Whether the genes above are related to low pretreatment platelet count needs further studies.

Previous studies have reported that PF can cause thrombocytopenia^44^ and has more hematologic toxicity.^45^ The CIT incidence rate of PF was 4.52%–8% in previous clinical trials.^46^, ^47^ In our study, more patients were included, and we found a much higher incidence rate of 10.17%. This was the first time that PF was reported as a risk factor for serious CIT. We found that GP regimen was a risk factor for CIT, which was consistent with the previous study.^48^ We found that TP regimen was also a risk factor for CIT whose OR was lower than PF. It is interesting that the three‐regimen chemotherapy, TPF, has less toxicity on thrombocytes or megakaryocytes. Further research is required.

It should be noted that we only included patients with NPC from one center, and whether the conclusions could fit other cancers requires further study. Further research is needed to determine whether the results could be applied to patients with pretreatment WBC count <4 × 10^9^/L or PLT count <100 × 10^9^/L.

## 5 CONCLUSION

Patients who experienced serious thrombocytopenia within 120 days from the start of treatment had a worse prognosis. Chemotherapy regimens of GP, PF, TP, LDH, K^+^, RBC, PLT count, and eGFR were predictors of serious CIT. Further studies are needed to explore the mechanism of the association between predictors and CIT and to prophylactically prevent CIT.

## AUTHOR CONTRIBUTIONS

**Lu‐Lu Zhang:** Conceptualization (lead); data curation (lead); formal analysis (lead); investigation (lead); methodology (lead); resources (lead); visualization (lead); writing – original draft (lead). **Xi Chen:** Methodology (equal); resources (equal). **Ying‐Ying Huang:** Resources (equal). **Chi‐Xiong Liang:** Resources (equal). **Meng‐Yun Qiang:** Resources (equal). **Zhuo‐Chen Cai:** Resources (supporting). **Ze‐Jiang Zhan:** Resources (supporting). **Ying Deng:** Resources (supporting). **Jia‐Yu Zhou:** Resources (supporting). **Hao‐Yang Huang:** Resources (supporting). **Xiang Guo:** Funding acquisition (equal); resources (equal). **Xing Lv:** Conceptualization (equal); funding acquisition (equal); supervision (lead); writing – review and editing (lead).

## FUNDING INFORMATION

This work was supported by the National Natural Science Foundation of China (Nos. 81872375 and 82172863) and the Natural Science Foundation of Guangdong Province (2021A1515010118).

## CONFLICT OF INTEREST STATEMENT

The authors have no conflict of interest to declare.

## ETHICS STATEMENT

This study was approved by the ethics committee of Sun Yat‐sen University Cancer Center (approval number B2022‐684‐01).

## PATIENT CONSENT STATEMENT

Written informed consent was not required because it was a retrospective study. The study was conducted in accordance with the Declaration of Helsinki.

## Data Availability

The key clinical data has been deposited in the Research Data Deposit (www.researchdata.org.cn), with the approval RDD number of RDDA2023333066.

## References

[1] Sung H , Ferlay J , Siegel RL , et al. Global cancer statistics 2020: GLOBOCAN estimates of incidence and mortality worldwide for 36 cancers in 185 countries. CA Cancer J Clin. 2021;71(3):209‐249. doi:10.3322/caac.21660 33538338

[2] Chen YP , Chan ATC , Le QT , Blanchard P , Sun Y , Ma J . Nasopharyngeal carcinoma. Lancet. 2019;394(10192):64‐80. doi:10.1016/s0140-6736(19)30956-0 31178151

[3] Chen YP , Ismaila N , Chua MLK , et al. Chemotherapy in combination with radiotherapy for definitive‐intent treatment of stage II–IVA Nasopharyngeal carcinoma: CSCO and ASCO guideline. J Clin Oncol. 2021;39(7):840‐859. doi:10.1200/jco.20.03237 33405943

[4] Adelstein DJ , Saxton JP , Lavertu P , et al. A phase III randomized trial comparing concurrent chemotherapy and radiotherapy with radiotherapy alone in resectable stage III and IV squamous cell head and neck cancer: preliminary results. Head Neck. 1997;19(7):567‐575. doi:10.1002/(sici)1097‐0347(199710)19:7<567::aid‐hed2>3.0.co;2‐5 9323144 10.1002/(sici)1097-0347(199710)19:7<567::aid-hed2>3.0.co;2-5

[5] Adelstein DJ , Li Y , Adams GL , et al. An intergroup phase III comparison of standard radiation therapy and two schedules of concurrent chemoradiotherapy in patients with unresectable squamous cell head and neck cancer. J Clin Oncol. 2003;21(1):92‐98. doi:10.1200/jco.2003.01.008 12506176

[6] Wendt TG , Grabenbauer GG , Rödel CM , et al. Simultaneous radiochemotherapy versus radiotherapy alone in advanced head and neck cancer: a randomized multicenter study. J Clin Oncol. 1998;16(4):1318‐1324. doi:10.1200/jco.1998.16.4.1318 9552032

[7] Mac Manus M , Lamborn K , Khan W , Varghese A , Graef L , Knox S . Radiotherapy‐associated neutropenia and thrombocytopenia: analysis of risk factors and development of a predictive model. Blood. 1997;89(7):2303‐2310. 9116273

[8] Weycker D , Hatfield M , Grossman A , et al. Risk and consequences of chemotherapy‐induced thrombocytopenia in US clinical practice. BMC Cancer. 2019;19(1):151. doi:10.1186/s12885-019-5354-5 30764783 PMC6376753

[9] Kilpatrick K , Shaw JL , Jaramillo R , et al. Occurrence and management of thrombocytopenia in metastatic colorectal cancer patients receiving chemotherapy: secondary analysis of data from prospective clinical trials. Clin Colorectal Cancer. 2021;20(2):170‐176. doi:10.1016/j.clcc.2020.10.004 33281065

[10] Elting LS , Cantor SB , Martin CG , et al. Cost of chemotherapy‐induced thrombocytopenia among patients with lymphoma or solid tumors. Cancer. 2003;97(6):1541‐1550. doi:10.1002/cncr.11195 12627519

[11] Goldberg GL , Gibbon DG , Smith HO , DeVictoria C , Runowicz CD , Burns ER . Clinical impact of chemotherapy‐induced thrombocytopenia in patients with gynecologic cancer. J Clin Oncol. 1994;12(11):2317‐2320. doi:10.1200/jco.1994.12.11.2317 7964946

[12] U.S. Department of Health And Human Services NIoH, National Cancer Institute . Common Terminology Criteria for Adverse Events (CTCAE) Version 5.0 . Available from https://ctep.cancer.gov/protocolDevelopment/electronic_applications/docs/CTCAE_v5_Quick_Reference_5x7.pdf

[13] Yoshida K , Bartel A . tableone:Create 'Table 1' to Describe Baseline Characteristics With or Without Propensity Score Weights . 2022. https://CRAN.R‐project.org/package=tableone

[14] Ho D , Imai K , King G , Stuart EA . MatchIt: nonparametric preprocessing for parametric causal inference. J Stat Softw. 2011;42(8):1‐28. doi:10.18637/jss.v042.i08

[15] Kassambara A , Kosinski M , Biecek P . survminer: Drawing Survival Curves using 'ggplot2' . 2021. https://CRAN.R‐project.org/package=survminer

[16] Therneau T . A Package for Survival Analysis in R . 2022. https://CRAN.R‐project.org/package=survival

[17] Frank E , Harrell J . rms: Regression Modeling Strategies . 2022. https://CRAN.R‐project.org/package=rms

[18] Robin X , Turck N , Hainard A , et al. pROC: an open‐source package for R and S+ to analyze and compare ROC curves. BMC Bioinform. 2011;12:77. doi:10.1186/1471-2105-12-77 PMC3068975 21414208

[19] Wan XB , Wei L , Li H , et al. High pretreatment serum lactate dehydrogenase level correlates with disease relapse and predicts an inferior outcome in locally advanced nasopharyngeal carcinoma. Eur J Cancer. 2013;49(10):2356‐2364. doi:10.1016/j.ejca.2013.03.008 23541571

[20] Inker LA , Schmid CH , Tighiouart H , et al. Estimating glomerular filtration rate from serum creatinine and cystatin C. N Engl J Med. 2012;367(1):20‐29. doi:10.1056/NEJMoa1114248 22762315 PMC4398023

[21] Poikonen‐Saksela P , Lindman H , Sverrisdottir A , et al. Leukocyte nadir as a predictive factor for efficacy of adjuvant chemotherapy in breast cancer. Results from the prospective trial SBG 2000–1. Acta Oncol. 2020;59(7):825‐832. doi:10.1080/0284186x.2020.1757149 32347139

[22] Tan X , Wen Q , Wang R , Chen Z . Chemotherapy‐induced neutropenia and the prognosis of colorectal cancer: a meta‐analysis of cohort studies. Expert Rev Anticancer Ther. 2017;17(11):1077‐1085. doi:10.1080/14737140.2017.1380521 28910204

[23] Daniele G , Arenare L , Scambia G , et al. Prognostic role of chemotherapy‐induced neutropenia in first‐line treatment of advanced ovarian cancer. A pooled analysis of MITO2 and MITO7 trials. Gynecol Oncol. 2019;154(1):83‐88. doi:10.1016/j.ygyno.2019.04.012 31029508

[24] Xu C , Yang SP , Zhang Y , et al. Neutropenia during the first cycle of induction chemotherapy is prognostic for poor survival in locoregionally advanced nasopharyngeal carcinoma: a real‐world study in an endemic area. Cancer Res Treat. 2018;50(3):777‐790. doi:10.4143/crt.2017.255 28745036 PMC6056978

[25] Bogani G , Sabatucci I , Maltese G , et al. Chemotherapy‐related leukopenia as a biomarker predicting survival outcomes in locally advanced cervical cancer. Eur J Obstet Gynecol Reprod Biol. 2017;208:41‐45. doi:10.1016/j.ejogrb.2016.11.017 27888705

[26] Abraham JE , Hiller L , Dorling L , et al. A nested cohort study of 6,248 early breast cancer patients treated in neoadjuvant and adjuvant chemotherapy trials investigating the prognostic value of chemotherapy‐related toxicities. BMC Med. 2015;13:306. doi:10.1186/s12916-015-0547-5 26715442 PMC4693418

[27] Di Maio M , Gridelli C , Gallo C , et al. Chemotherapy‐induced neutropenia and treatment efficacy in advanced non‐small‐cell lung cancer: a pooled analysis of three randomised trials. Lancet Oncol. 2005;6(9):669‐677. doi:10.1016/s1470-2045(05)70255-2 16129367

[28] Fortin A , Wang CS , Vigneault E . Effect of pretreatment anemia on treatment outcome of concurrent radiochemotherapy in patients with head and neck cancer. Int J Radiat Oncol Biol Phys. 2008;72(1):255‐260. doi:10.1016/j.ijrobp.2008.04.079 18632214

[29] Chua DT , Sham JS , Choy DT . Prognostic impact of hemoglobin levels on treatment outcome in patients with nasopharyngeal carcinoma treated with sequential chemoradiotherapy or radiotherapy alone. Cancer. 2004;101(2):307‐316. doi:10.1002/cncr.20366 15241828

[30] Sher DJ , Posner MR , Tishler RB , et al. Relationship between radiation treatment time and overall survival after induction chemotherapy for locally advanced head‐and‐neck carcinoma: a subset analysis of TAX 324. Int J Radiat Oncol Biol Phys. 2011;81(5):e813‐e818. doi:10.1016/j.ijrobp.2010.12.005 21300455

[31] Cannon DM , Geye HM , Hartig GK , et al. Increased local failure risk with prolonged radiation treatment time in head and neck cancer treated with concurrent chemotherapy. Head Neck. 2014;36(8):1120‐1125. doi:10.1002/hed.23419 23804248

[32] Shaikh T , Handorf EA , Murphy CT , Mehra R , Ridge JA , Galloway TJ . The impact of radiation treatment time on survival in patients with head and neck cancer. Int J Radiat Oncol Biol Phys. 2016;96(5):967‐975. doi:10.1016/j.ijrobp.2016.08.046 27869097 PMC5147736

[33] Liu SL , Sun XS , Yan JJ , et al. Optimal cumulative cisplatin dose in nasopharyngeal carcinoma patients based on induction chemotherapy response. Radiother Oncol. 2019;137:83‐94. doi:10.1016/j.radonc.2019.04.020 31078941

[34] Wen DW , Li ZX , Chen FP , et al. Individualized cumulative cisplatin dose for locoregionally‐advanced nasopharyngeal carcinoma patients receiving induction chemotherapy and concurrent chemoradiotherapy. Oral Oncol. 2020;107:104675. doi:10.1016/j.oraloncology.2020.104675 32361563

[35] Razzaghdoust A , Mofid B , Zangeneh M . Predicting chemotherapy‐induced thrombocytopenia in cancer patients with solid tumors or lymphoma. J Oncol Pharm Practice. 2020;26(3):587‐594. doi:10.1177/1078155219861423 31315547

[36] Meng QH , Wagar EA . Pseudohyperkalemia: a new twist on an old phenomenon. Crit Rev Clin Lab Sci. 2015;52(2):45‐55. doi:10.3109/10408363.2014.966898 25319088

[37] Kimura M , Lu X , Skurnick J , et al. Potassium chloride supplementation diminishes platelet reactivity in humans. Hypertension. 2004;44(6):969‐973. doi:10.1161/01.HYP.0000147660.58694.6f 15505115

[38] Lu R , Lin Q , Chen S , Ye X . Chemotherapy‐induced thrombocytopenia and platelet transfusion in patients with diffuse large B‐cell lymphoma. Transl Cancer Res. 2020;9(3):1640‐1651. doi:10.21037/tcr.2020.01.64 35117512 PMC8798421

[39] Hu J , Tang L , Cheng Y , Liu A , Huang L . Risk analysis of severe thrombocytopenia in nasopharyngeal carcinoma during concurrent radio‐chemotherapy. Front Oncol. 2021;11:754624. doi:10.3389/fonc.2021.754624 35186708 PMC8847770

[40] Blay JY , Le Cesne A , Mermet C , et al. A risk model for thrombocytopenia requiring platelet transfusion after cytotoxic chemotherapy. Blood. 1998;92(2):405‐410. 9657738

[41] Alidina A , Gaffar A , Hussain F , et al. Survival data and prognostic factors seen in Pakistani patients with esophageal cancer. Ann Oncol. 2004;15(1):118‐122. doi:10.1093/annonc/mdh014 14679130

[42] Chen YP , Chen C , Mai ZY , et al. Pretreatment platelet count as a predictor for survival and distant metastasis in nasopharyngeal carcinoma patients. Oncol Lett. 2015;9(3):1458‐1466. doi:10.3892/ol.2015.2872 25663931 PMC4314978

[43] Björn N , Sigurgeirsson B , Svedberg A , et al. Genes and variants in hematopoiesis‐related pathways are associated with gemcitabine/carboplatin‐induced thrombocytopenia. Pharmacogenomics J. 2020;20(2):179‐191. doi:10.1038/s41397-019-0099-8 31616045

[44] Ke LR , Xia WX , Qiu WZ , et al. Safety and efficacy of lobaplatin combined with 5‐fluorouracil as first‐line induction chemotherapy followed by lobaplatin‐radiotherapy in locally advanced nasopharyngeal carcinoma: preliminary results of a prospective phase II trial. BMC Cancer. 2017;17(1):134. doi:10.1186/s12885-017-3080-4 28202000 PMC5311839

[45] Kong TW , Chang SJ , Paek J , et al. Comparison of concurrent chemoradiation therapy with weekly cisplatin versus monthly fluorouracil plus cisplatin in FIGO stage IIB‐IVA cervical cancer. J Gynecol Oncol. 2012;23(4):235‐241. doi:10.3802/jgo.2012.23.4.235 23094126 PMC3469858

[46] Posner MR , Hershock DM , Blajman CR , et al. Cisplatin and fluorouracil alone or with docetaxel in head and neck cancer. N Engl J Med. 2007;357(17):1705‐1715. doi:10.1056/NEJMoa070956 17960013

[47] Lv X , Cao X , Xia WX , et al. Induction chemotherapy with lobaplatin and fluorouracil versus cisplatin and fluorouracil followed by chemoradiotherapy in patients with stage III–IVB nasopharyngeal carcinoma: an open‐label, non‐inferiority, randomised, controlled, phase 3 trial. Lancet Oncol. 2021;22(5):716‐726. doi:10.1016/s1470-2045(21)00075-9 33857411

[48] Ten Berg MJ , van den Bemt PM , Shantakumar S , et al. Thrombocytopenia in adult cancer patients receiving cytotoxic chemotherapy: results from a retrospective hospital‐based cohort study. Drug Saf. 2011;34(12):1151‐1160. doi:10.2165/11594310-000000000-00000 22077503

